# Multi-nutrient supplement improves hormone ratio associated with cancer risk

**DOI:** 10.1186/1479-5876-11-252

**Published:** 2013-10-08

**Authors:** Anthony J Bazzan, George P Zabrecky, Andrew B Newberg

**Affiliations:** 1 Myrna Brind Center of Integrative Medicine, Thomas Jefferson University, 925 Chestnut Street, Suite 120, Philadelphia, PA 19107, USA

**Keywords:** Hormone, Nutrient, Cancer, Proliferative, Indole, Flavonoid

## Abstract

**Background:**

Gynecological cancers are among the most common in women and are directly related to a variety of hormonal factors. One potential risk factor associated with developing a gynecological malignancy is the ratio of two hormone metabolites, 2-Hydroxyestrone (2-HE) and 16alpha-Hydroxyestrone (16alpha-HE). A number of botanical constituents such as indoles, flavonoids, and resveratrol have been shown to have a favorable effect on the metabolic pathways that affect this ratio. The present study was designed to evaluate if a multi-nutrient supplement containing targeted botanical constituents would affect the 2-HE/16 alpha-HE ratio in middle-aged women.

**Methods:**

A retrospective analysis was performed on 76 female patients (mean age 54 years) who received 2-HE/16 alpha-HE ratio assessments at two separate time points. The ratio assessment was part of standard care for women who presented with risk indicators associated with a high proliferative state. All patients who completed pre and post assessments were included. Sixty-five of the patients received a multi-nutrient supplement, Lucentia Peak®, during the study period. Eleven patients chose not to take the supplement, but did receive ratio assessments at similar time points as the treatment group, allowing for between group comparisons. Paired t-tests were used to compare the changes in the 2-HE and 16alpha-HE measures as well as their ratio, both within groups and between groups.

**Results:**

The results demonstrated a significant increase in the 2-HE/16alpha-HE ratio in the treated group (pre 0.38 to post 0.57, p<0.0001), and was significantly different (p=0.02) compared to the change in the control group (pre 0.65 to post 0.64). This change appears to be mediated primarily by an increase in the 2-HE level. Individually, 54 patients given Lucentia Peak® had increased ratios while 11 patients had a decrease. In the control group, 3 patients had an increase in their ratio and 8 patients had a decrease.

**Conclusions:**

The results demonstrated that women receiving the Lucentia Peak® multi-nutrient supplement had significant increases in their 2-HE:16alpha-HE ratio, which appears to be mediated primarily by increasing the 2-HE levels. These results suggest further research on phytonutrients that might positively affect estrogen metabolism is warranted.

## Background

Gynecological cancers are among the most common in women and are directly related to a variety of hormones. One potential risk for developing a gynecological malignancy is the relative ratio of two hormone metabolites, 2-Hydroxyestrone (2-HE) and 16alpha-Hydroxyestrone (16alpha-HE). Several early studies showed no significant difference in this 2-HE/16alpha-HE ratio between women currently with or without breast cancer [1-3]. However, more recent studies, including a longitudinal evaluation, showed that a higher ratio lowers the risk of developing future gynecological cancers [4,5]. Approaches that positively affect this ratio could have important implications for patients at risk of developing gynecological cancers.

Several naturally occurring, plant-derived compounds have been investigated for increasing the 2-HE/16alpha-HE ratio. Indoles may help increase this ratio; in particular, indole-3-carbinol (I3C), a natural compound derived by hydrolysis from glycobrassicin produced in cruciferous vegetables such as cabbage, broccoli, and Brussels sprouts, and its natural diindole condensation product 3-3’-diindolylmethane (DIM) [6]. Several studies provide evidence that both of these indoles can improve the 2-HE/16alpha-HE ratio when given in the appropriate dose [7]. DIM, can induce estrogenic responses through a ligand-independent activation of estrogen receptors or disrupt estrogen responsiveness through its interaction with the aryl hydrocarbon receptor [8,9].

Bioflavonoids have also been found to have anti-proliferative properties [10-12] that are mediated by several mechanisms. For example, bioflavonoids can interfere with several different free radical–producing systems and can also increase the function of the endogenous antioxidants [13] and can also inhibit cell proliferation and angiogenesis [14,15]. However, it is not clear whether bioflavonoids specifically alter the 2-HE/16alpha-HE ratio. Another compound, resveratrol, has been shown to regulate estrogen effects also possibly conferring a beneficial effect with regard to the development of malignancy [16-18]. Several other herbal supplements might have beneficial effects as well. For example, icariin, a prenyl flavonoid derivative from Epimedium Genus has been shown to induce cell cycle arrest in breast cancer cells [19] and also has estrogenic effects [20]. Red clover has been shown to possess antioxidant and antiinflammatory activities, as well as inhibit angiogenesis and displaying anti-cancer properties [21]. Black currant extracts have been shown to have a variety of phytochemicals that have anti-proliferative effects via multiple cellular mechanisms [22].

These studies and others have led to the development of multi-nutrient supplements that blend compounds such as these to help improve the hormonal ratio and reduce the proliferative state. The goal of the present study was to retrospectively evaluate the effects of a multi-nutrient supplement designed for this purpose.

## Methods

All patients in a university hospital out-patient setting who received 2-HE/16alpha-HE assessments at two distinct time points between 2010 and 2012 were included in this study. We retrospectively evaluated the hormone metabolism ratio of 65 patients (mean age 54±9 years) receiving the multi-nutrient supplement, Lucentia Peak®, for dietary support and preventive care. Since this was a retrospective evaluation of existing clinical data, this study was deemed exempt from review by the Institution Review Board (IRB) at Thomas Jefferson University and was administratively approved as per IRB guidelines. The supplement contains a combination of resveratrol, diindolylmethane, epimedium, eucommia, passion flower, suma, damiana, red clover, black currant extract, white button mushroom, and also vitamin E and C, calcium, and magnesium. The exact contents are provided in Table 1. We compared these results to 11 patients (mean age 54±5 years) who chose not to take the supplement, but were willing to receive a follow-up ratio assessment as part of overall clinical care. This formed the control group for the comparison. The mean duration of treatment was 196±175 days. The mean time between evaluations for the treatment group was 288±343 days and for the control group was 891±531 days. A paired t-test was used to compare the changes in the 2-HE and 16alpha-HE measures as well as their ratio. In addition, comparison between the ratio changes for the two groups was also performed. Serum measures of the 2-HE/16alpha-HE ratio were used in this study (27).

**Table 1:** Supplement contents given as the amount of compound in one serving (4 capsules)

Calcium D- Glucarate	150 mg
Magnesium Citrate	50 mg
Resveratrol	20 mg
Poly-ionic Saccharide Complex	175 mg
(Zehntose™)	
Red Clover Extract	125 mg
(Standardized to 8% isoflavone)	
BioResponse DIM® Complex	75 mg
(Diindolylmethane)	
Epimedium (Stem and Leaf Extract)	250 mg
(Standardized to 10% Icariin)	
Eucommia Bark Extract	100 mg
(Standardized to 40% Chlorogenic Acid)	
Passion Flower (Leaf) Extract	75 mg
(Standardized to 3.5% as Isovitexin)	
Suma (Root) Extract	60 mg
Damiana (Leaf) Extract	40 mg
Black Currant Extract	120 mg
White Button Mushroom	100 mg

## Results

The results are shown in Table 2 and demonstrate a significant increase in the 2-HE levels and in the 2-HE/16alpha-HE ratio in the treated group (p < 0.0001), but no change to either of these measures in the untreated group. On an individual basis, 54 patients given Lucentia Peak had an increase in their 2-HE/16alpha-HE ratio (i.e. improvement), while 11 patients had a decrease (worsening). In the control group, 3 patients had an increase in their 2-HE/16alpha-HE ratio and 8 patients had a decrease. Overall, the treated group had a mean increase in the 2-HE/16alpha-HE ratio of 18 points (50%) while the control group had a decrease of 1 point. This difference between the treated and control group change in the ratio was statistically significant (p=0.02) based on a t-test comparing the change between the treated and control groups.

**Table 2:** Comparison of initial and follow up measures of the 2-HE/16alpha-HE ratio in those patients treated with Lucentia Peak® and those patients who were untreated control subjects

**Peak group**	**2-HE**	**16alpha-HE**	**Ratio**	**Control group**	**2-HE**	**16alpha-HE**	**Ratio**
**Pre Mean**	170.0	464.8	0.38	**Initial Mean**	321.8	525.4	0.65
**Pre SD**	96.2	137.5	0.21	**Initial SD**	191.4	346.2	0.34
**Post Mean**	262.4	490.0	0.57	**Follow up Mean**	278.7	479.2	0.64
**Post SD**	117.7	173.0	0.27	**Follow up SD**	131.1	174.6	0.33
**p-value**	< 0.0001	0.1380	< 0.0001		0.301	0.290	0.474

## Discussion

The results from the current study demonstrated that women who received the Lucentia Peak® multi-nutrient supplement had substantial improvements in their 2-HE:16alpha-HE ratio, and this appears to be mediated primarily by increasing the 2-HE levels. These results were comparable to another study that gave indole-3-carbinol to women at high risk for breast cancer [16]. In that study, 17 women were evaluated and treated with increasing doses of indole-3-carbinol for 4 weeks. Patients receiving 800 mg per day demonstrated elevation in the CYP1A2 activity, which mirrored a 66% increase in the urinary 2-HE/16alpha-HE ratio in response to I3C. In that study, the maximal increase was observed with the 400 mg daily of I3C, with no further increase found at 800 mg daily. In a related study, Wong et al. demonstrated in 57 women that 300 mg per day of I3C significantly increased the 2-HE/16alpha-HE ratio [7]. Additionally, the other indole, DIM has antitumorogenesis effects [23] in breast cancer. And a small placebo controlled study of 19 women given 108 mg DIM/day for 30 days showed a significant increase in levels of 2-HE (P=0. 020) even though the limited sample revealed a nonsignificant increase of 47% in the 2-OHE/16alpha-OHE ratio (P=0.059) [24].

Studies of the other compounds in Lucentia Peak® also have revealed potential anti-cancer effects mediated either through hormonal effects or other physiological processes. For example, resveratrol has been shown to inhibit estrogen-DNA adduct formation via its antioxidant function and to do so approximately 50% more effectively than N-acetyl cysteine [25]. Icaritin, a prenyl flavonoid derivative from Epimedium Genus has been shown to induce cell cycle arrest in breast cancer cell lines *in vitro*[19] and to have estrogenic effects in rat studies [20]. Other compounds with estrogenic effects such as red clover are frequently used by women with breast cancer [26]. However, their effectiveness is not established. Finally, several components in Lucentia Peak® also may alter the expression of various inflammatory biomarkers. Such anti-inflammatory properties might play an important role in the potential anti-tumorogenesis effects of this multi-nutrient supplement [11,13,21,22].

Limitations of this study include that it was a retrospective evaluation. Future randomized, prospective and controlled trials will be necessary to better determine whether multi-nutrient supplements help reduce the 2-HE/16alpha-HE ratio, and subsequently, the risk of gynecological cancers. A placebo control would be particularly important, especially in those patients with low 2-HE/16alpha-HE ratios so that it can be more clearly determined whether the supplement has a definitive effect. Selection bias is a consideration since the treated group generally had lower values for the 2-HE/16alpha-HE ratio and therefore may have been given more encouragement by their treating physician to utilize the supplement for this purpose. However, it is noted that the 2-HE/16alpha-HE ratios for the untreated group did not change substantially.

## Conclusions

Overall, this study showed that women with a low 2-HE/16alpha-HE ratio who were given Lucentia Peak® experienced an increase in the ratio, specifically related to an increase in the measure of 2-HE. Given the potential reduction in the risk of gynecological cancer in patients having a higher level of 2-HE and the hormone ratio, this study is an important step in evaluating a dietary supplements that may have implications for women’s preventive health.

## Competing interest

AJB is a shareholder in the company that distributes the product, Lucentia Peak®. GPZ has no conflict of interest to report. ABN has no conflict of interest to report.

## Authors’ contributions

AJB treated the patients, helped obtain and evaluate the data, and drafted the manuscript. GZ helped with data analysis and drafting the manuscript. ABN participated in the design of the study and performed the statistical analysis as well as drafting the manuscript. All authors read and approved the final manuscript.
